# Gaofen-3 PolSAR Image Classification via XGBoost and Polarimetric Spatial Information

**DOI:** 10.3390/s18020611

**Published:** 2018-02-17

**Authors:** Hao Dong, Xin Xu, Lei Wang, Fangling Pu

**Affiliations:** 1 School of Electronic Information, Wuhan University, Wuhan 430072, China; donghao@whu.edu.cn (H.D.); wanglei2016@whu.edu.cn (L.W.); 2 Collaborative Innovation Center of Geospatial Technology, Wuhan University, Wuhan 430079, China; flpu@whu.edu.cn

**Keywords:** Gaofen-3 (GF-3), polarimetric synthetic aperture radar (PolSAR), image classification, XGBoost, spatial information

## Abstract

The launch of the Chinese Gaofen-3 (GF-3) satellite will provide enough synthetic aperture radar (SAR) images with different imaging modes for land cover classification and other potential usages in the next few years. This paper aims to propose an efficient and practical classification framework for a GF-3 polarimetric SAR (PolSAR) image. The proposed classification framework consists of four simple parts including polarimetric feature extraction and stacking, the initial classification via XGBoost, superpixels generation by statistical region merging (SRM) based on Pauli RGB image, and a post-processing step to determine the label of a superpixel by modified majority voting. Fast initial classification via XGBoost and the incorporation of spatial information via a post-processing step through superpixel-based modified majority voting would potentially make the method efficient in practical use. Preliminary experimental results on real GF-3 PolSAR images and the AIRSAR Flevoland data set validate the efficacy and efficiency of the proposed classification framework. The results demonstrate that the quality of GF-3 PolSAR data is adequate enough for classification purpose. The results also show that the incorporation of spatial information is important for overall performance improvement.

## 1. Introduction

The Chinese Gaofen-3 (GF-3) satellite carrying a synthetic aperture radar (SAR) sensor, was launched on 10 August 2016. With a powerful imaging capability of 12 modes, GF-3 can provide polarimetric SAR (PolSAR) images with a nominal resolution of 8 m. By transmitting and receiving horizontal and vertical waves, PolSAR data is able to capture four polarization channels, helpful for physical understanding and mechanism interpretation regarding land surface [1]. Many applications such as crop monitoring, biomass estimation, target detection, land cover and land use classification, can be undertaken by choosing appropriate GF-3 SAR data. Among the applications, the classification of PolSAR image plays an important role in PolSAR image analysis. Actually, land cover classification on a real whole PolSAR image is the main task in our cooperation with the National Disaster Reduction Center of China (NDRCC). How to find an efficient framework to classify an entire PolSAR image quickly and accurately is the main concern in practical use.

In the last two decades, researchers have developed many PolSAR image classification algorithms. In general, these algorithms can be divided into three categories called statistical model-based algorithms [2,3,4], scattering mechanism analysis-based algorithms [5,6,7], and algorithms combining standard classifiers and polarimetric features [8]. Many works focus on the third type among these approaches. More and more complicated and efficient classifiers or features are integrated into the PolSAR image classification framework to improve accuracy [9,10,11,12]. For example, many neural network(NN)-based methods are proposed and they have reported very excellent results in recent years [11,13,14,15]. However, even though accuracy is improved, most classification algorithms and procedures of complicated feature extraction are of high complexity and time-consuming, which makes them impractical to process an entire PolSAR image. Moreover, enough labeled samples for NN-based classifiers are not always available. Therefore, an efficient classifier with high accuracy and low time cost is preferable in practical use, which also explains why tree-based classifiers are still welcome in remotely sensed image analysis [16,17,18,19,20]. In Ref. [16], QUEST (quick, unbiased, efficient, statistical tree [21]) was used as a decision tree (DT) tool to implement the classification. The decision tree was very efficient and provided clear split rules that can be easily interpreted according to the physical understanding of used features. Du et al. [17] used random forest (RF) and rotation forest to investigate polarimetric-spatial features in PolSAR image classification and found that rotation forest performed better while RF was much faster [17]. Pradhan et al. [18] compared the performance of several classification techniques for extracting urban areas. It turned out that the rule-based classifier and DT distinguished land cover classes better than support vector machine (SVM) and K-nearest neighbor (KNN) [18]. In [19,20], hybrid DT and RF were used as classifiers, separately, and showed comparable results. Previous work has shown that the tree-based classifier is very efficient and popular in practical use.

Polarimetric features are often associated with the scattering mechanism of terrain scatterers and represent the information embedded in PolSAR data. The quality of input features basically determines the classification performance of employed classifiers. Besides polarimetric information, spatial information is another important factor that affects classification performance and has been paid more and more attentions. The introduction of spatial features in PolSAR image analysis is inspired by the remarkable improvements resulting from the complementarity between spectral and spatial features in optical image classification [17]. Markov random fields (MRF) modeling, texture information and region-based or object-based algorithms are the three main methodological approaches that can be employed to incorporate spatial information into image analysis [22,23]. Du et al. combined spatial features (consisting of textural metrics and morphological profiles) with polarimetric features for PolSAR image classification [17]. In our previous work, spatial information and statistical model-based data fidelity were incorporated into the classification framework via MRF [24]. In Ref. [25], superpixels were generated by a modified linear iterative clustering algorithm to incorporate spatial context. Another potential benefit due to the incorporation of spatial information is to reduce speckles, which are very common in PolSAR images and adversely affect the classification performance.

To make the classification method more practical in use and incorporate spatial information at each image pixel, we select a recently proposed tree-based classification framework called XGBoost [26] for initial image classification. Spatial information is incorporated via superpixels generated by the statistical region merging (SRM) method[27]. After superpixels are generated, the label of a superpixel is determined in a post-processing step through a modified majority voting strategy, which runs quickly and turns out to be efficient. The remainder of this paper is organized as follows. In Section 2, we introduce the adopted features, related classifiers and the proposed classification framework. In Section 3, we describe the experimental data sets and analyze the classification results obtained on real GF-3 PolSAR images. Additional discussions are presented in Section 4. Conclusions are drawn in Section 5.

## 2. Method

### 2.1. Polarimetric Features

Providing more information for land cover classification, PolSAR data can be represented by a scattering matrix **S**, a covariance matrix **C** or a coherency matrix **T** [1]. ${\left|HH\right|}^{2}$, ${\left|HV\right|}^{2}$, and ${\left|VV\right|}^{2}$ represent the scattered power of three channels and directly reflect the type of scattering media. With an assumption of reciprocal condition $S_{HV}=S_{VH}$, the coherency matrix can be expressed by $T_{3}$, of which the diagonal elements are $T_{11}$, $T_{22}$, and $T_{33}$, respectively.

(1) $$T_{11}=0.5*|S_{HH}+S_{VV}{|}^{2},\;T_{22}=0.5*|S_{HH}-S_{VV}{|}^{2},\;T_{33}=2{\left|S_{HV}\right|}^{2}$$

Another useful polarimetric parameter is the circular polarization correlation coefficient ρ [28], which can be expressed as

(2) $$\rho =\frac{<S_{RR}S_{LL}^{*}>}{\sqrt{<|S_{RR}{|}^{2}><{\left|S_{LL}\right|}^{2}>}}$$

Man-made targets such as buildings exhibit low surface roughness and a high value of |ρ| [29]. Many other techniques also have been proposed to decompose PolSAR data and most of these can be divided into two categories [30]. One category is based on an eigenvector-based decomposition proposed by Cloude [5]. Three eigenvector-based parameters of entropy *H*, scattering angle α, and anisotropy *A* can classify a pixel by scattering mechanisms.

(3) $$\begin{array}{c} H=-\sum_{i=1}^{3}p_{i}\log_{3}p_{i},\alpha =\sum_{i=1}^{3}p_{i}\alpha_{i},A=\frac{\lambda_{2}-\lambda_{3}}{\lambda_{2}+\lambda_{3}} \end{array}$$

where $p_{i}$ correspond to the pseudo-probabilities from the eigenvalues, $\lambda_{i}$ are the sorted eigenvalues of $T_{3}$, and $\alpha_{i}$ represent scattering mechanism associated with each eigenvector. The other category is model-based decomposition such as Freeman decomposition, which models PolSAR data as the sum of surface, volume, and double-bounce scattering components under an assumption of reflection symmetry [6]. Yamaguchi et al. went further than the Freeman decomposition by introducing the helix component [7].

(4) $$span=YP_{S}+YP_{V}+YP_{D}+YP_{C}$$

However, it has been demonstrated that the Yamaguchi decomposition may generate negative powers due to overestimation in the volume scattering, which is obviously nonphysical [30]. Van Zyl et al. tackled the problem by introducing the nonnegative eigenvalue decomposition (NNED) and ensured nonnegative scattering powers of different scattering mechanisms [30].

In this paper, we extract 16 polarimetric features to represent the information embedded in GF-3 PolSAR data. Due to the relation between $T_{33}$ and ${\left|HV\right|}^{2}$, only $T_{33}$ is taken into consideration. Even though Van Zyl NNED and Yamaguchi decompositions reflect similar scattering mechanisms, we simultaneously use the two decompositions since the extraction procedures are different and Yamaguchi decomposition provides another helix component. Therefore, the total selected features consist of ${\left|HH\right|}^{2}$, ${\left|VV\right|}^{2}$, the diagonal elements of $T_{3}$($T_{11}$, $T_{22}$, $T_{33}$), |ρ|, three eigenvector-based parameters from H/α/A model (*H*, α, *A*), four components of Yamaguchi decomposition ($YP_{S}$, $YP_{V}$, $YP_{D}$, $YP_{C}$) and three components of Van Zyl NNED decomposition ($VP_{S}$, $VP_{V}$, $VP_{D}$).

### 2.2. XGBoost for PolSAR Image Classification

In PolSAR image classification, the goal is to determine the labels of unknown scattering cells or pixels based on a fine-trained classifier. The adopted XGBoost is short for “extreme gradient boosting” and designed to be a scalable machine learning system for tree boosting. The system runs much faster and gives state-of-the-art results on many problems from data mining challenges [26]. Given *n* labeled samples with *m* features $D=\left(x_{i},y_{i}\right)\left(|D\right|=n,x_{i}\in {ℜ}^{m},y_{i}\in ℜ)$, the tree ensemble method uses *K* additive functions to predict the label.

(5) $${\hat{y}}_{i}=\sum_{k=1}^{K}f_{k}\left(x_{i}\right),f_{k}\in F$$

where $F=\left\{f\left(x\right)=w_{q(x)}\right\}\left(q:{ℜ}^{m}\to T,w\in {ℜ}^{T}\right)$ is the space of regression trees. *q* represents the structure of each tree that has *T* leaves. Each $f_{k}$ corresponds to an independent tree structure *q* and leaf weights *w*. To learn the set of functions in the model, XGBoost minimizes the following regularized objective.

(6) $$L=\sum_{i}l\left({\hat{y}}_{i},y_{i}\right)+\sum_{k}\Omega \left(f_{k}\right),\;where\;\Omega \left(f\right)=\gamma T+\frac{1}{2}{\lambda ||w||}^{2}$$

Here *l* is the loss function and Ω is the regularized term. The regularized objective is slightly improved compared to previous gradient tree boosting algorithm. Let ${\hat{y}}_{i}^{\left(t\right)}$ be the prediction of the *i*-th instance at the *t*-th iteration. The ensemble model works better in an additive manner. Second-order approximation is used to speed up the optimization procedure.

(7) $$L^{\left(t\right)}=\sum_{i=1}^{n}l\left(y_{i},{\hat{y}}_{i}^{\left(t-1\right)}+f_{t}\left(x_{i}\right)\right)+\Omega \left(f_{t}\right)$$

(8) $$L^{t}≃\sum_{i=1}^{n}\left[l\left(y_{i},{\hat{y}}_{i}^{\left(t-1\right)}\right)+g_{i}f_{t}\left(x_{i}\right)+\frac{1}{2}h_{i}f_{t}^{2}\left(x_{i}\right)\right]+\gamma T+\frac{1}{2}\lambda \sum_{j=1}^{T}w_{j}^{2}$$

where $g_{i}$ and $h_{i}$ are first and second order gradient statistics on the loss function. For a fixed structure q(x), the optimal weight w and the corresponding optimal value can be calculated by evaluating the split candidates.

Besides the improvements in the regularized objective, several additional techniques are also used to further promote classification performance. Shrinkage and column/feature subsampling are used to prevent overfitting. Column/feature subsampling can also speed up computations of the algorithm. In order to find the best split efficiently, the algorithm visits feature values in sorted order to accumulate the gradient statistics in (8) and an approximate algorithm is summarized to avoid enumerating all possible splits greedily. In system design level, a block structure is used to reduce the cost of sorting, which is the most time consuming part of tree learning. Readers can refer to [26] for detailed information of XGBoost. In case of the mentioned advancements and excellent performance in practical use, XGBoost is adopted for PolSAR image to quickly generate the initial classification map.

### 2.3. Post-Processing via Superpixels and Majority Voting

**Figure 1:**
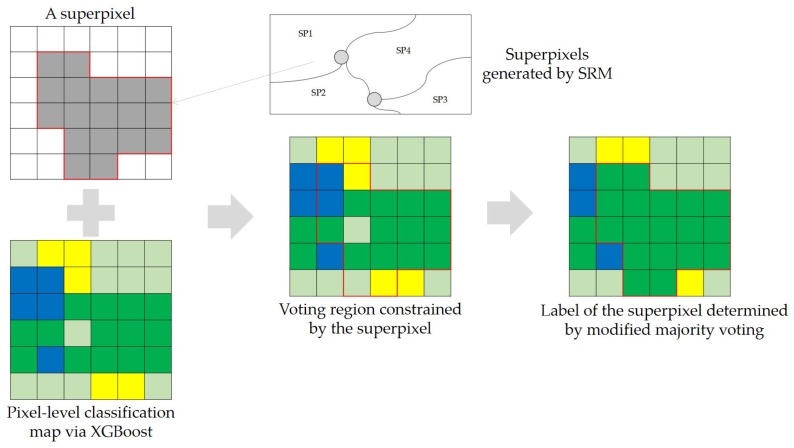
Incorporation of spatial information in the post-posting step via superpixels and modified majority voting. Class types are represented by corresponding colors.

To incorporate spatial information into the pixel-wise classification framework, we adopt the decision strategy of superpixel-based majority voting, which is similar to [31] except for the decision rule and the generation algorithm of superpixels. Superpixels can be generated via segmentation, which is another important application of PolSAR images. Many superpixel segmentation algorithms suitable for PolSAR data have been proposed by incorporating polarimetric information. Lang et al. improved SRM considering the characteristics of PolSAR data and made the generalized SRM suitable for single- or multi-dimensional SAR data [32]. Xiang et al. modified the simple linear iterative clustering(SLIC) and developed an adaptive superpixel generation procedure with local iterative clustering and spherically invariant random vector (SIRV) [33]. Wang et al. introduced two distance measures and an entropy rate method into the PolSAR image superpixel segmentation [34]. In this paper, statistical region merging is directly applied to the Pauli RGB image to generate superpixels for its rapidity [27,32]. The parameter *Q* in SRM quantifies the statistical complexity of a perfect scene $I^{*}$ and controls the coarseness of the segmentation. Conceptual simplicity and the ability of coping with significant noise corruption make SRM suitable for superpixel generation of PolSAR image. One way to use superpixels is to apply classification algorithms on superpixels directly. But it is inconvenient for users to select superpixels as training samples and may also decrease the number of available samples. In this paper, the post-processing procedure of superpixel-based majority voting is shown in Figure 1. The direct use of superpixel-based majority voting goes both ways. It may misclassify a superpixel if the majority is misclassified. To weaken such an effect, we modify the majority voting. For a given superpixel $sp_{j}$ with *n* pixels that are classified into *C* classes, we construct the normalized histogram $H=\{H_{1},H_{2},\cdots ,H_{C}\}$ of class labels and the sorted histogram $\mathrm{Hs}=\{H_{s1},H_{s2},\cdots ,H_{sC}\}$ in descending order. If $H_{s1}<=\sigma_{1}$ and $H_{s2}>=\sigma_{2}$, the pixels labeled s2 are kept and the remaining pixels are assigned as the majority label. Otherwise, the label with the highest frequency is assigned to all pixels within the superpixel $sp_{j}$.

(9) $$C_{sp_{j}}=\underset{i\in \{1,2,\cdots ,C\}}{\arg\;\max}\left(H_{i}\right)$$

**Figure 2:**
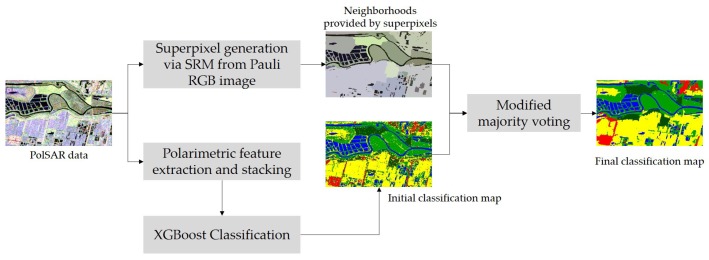
Workflow of the proposed method.

The proposed classification framework consists of four simple parts including polarimetric feature extraction and stacking, the initial classification via XGBoost, superpixels generation by SRM based on the Pauli RGB image, and label determination via a post-processing step through superpixel-based modified majority voting. The extended Lee Sigma filter is actually used to pre-process the original GF-3 PolSAR images for speckle filtering [35,36]. The workflow of the method is presented in Figure 2. Fast initial classification via XGBoost and the incorporation of spatial information in the post-processing step via superpixel-based modified majority voting, would potentially make the method efficient in practical use and promote the classification performance.

## 3. Experiments and Results Analysis

### 3.1. Experimental Data

**Figure 3:**
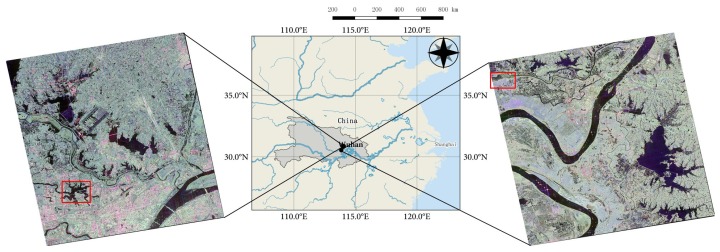
Geographic extent of the study area and Pauli RGB images of the GF-3 PolSAR data (left: image #1, right: image #2). The red rectangles indicate the locations of the sub-images that are cropped.

**Figure 4:**
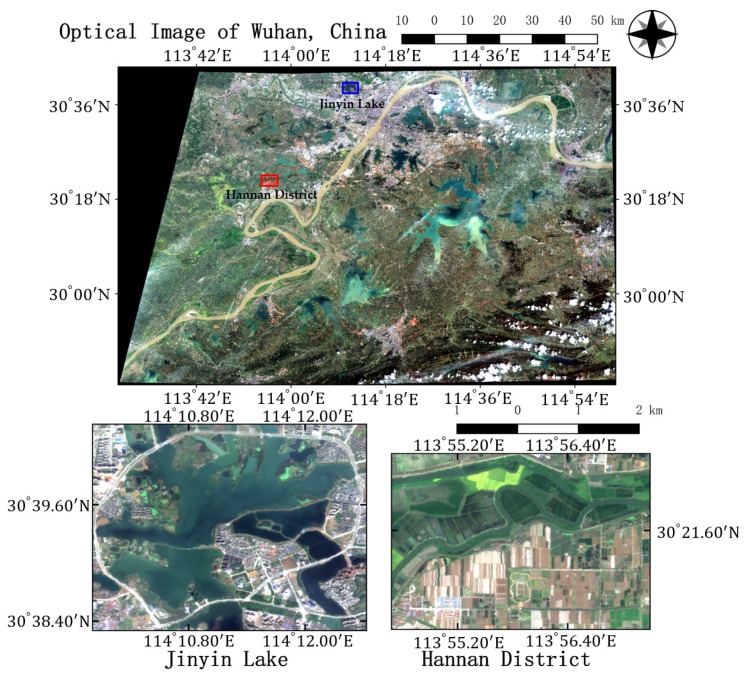
Optical image of the study area. Above is the mosaic Sentinel-2 optical image and the ROIs are masked by blue and red rectangles, respectively. On the bottom are the zoomed-in images of ROIs.

In this study, the city of Wuhan, which lies in central China, is chosen as a case study area. Two GF-3 single-look complex (SLC) PolSAR images with quad-polarized strip I (QPSI) mode are captured to evaluate the proposed algorithm. Both images are acquired with similar imaging parameters over Wuhan, China, as shown in Figure 3. The detailed data information is presented in Table 1. Two sub-images specified by the red rectangles in Figure 3 are cropped from the two whole images (images #1 and #2) and we name the sub-images data set A and data set B, respectively. Two optical images acquired by Sentinel-2 [37] on 26 March 2017 are also collected for identifying land cover classes. Figure 4 shows the mosaic image and the two regions of interest.

**Figure 5:**
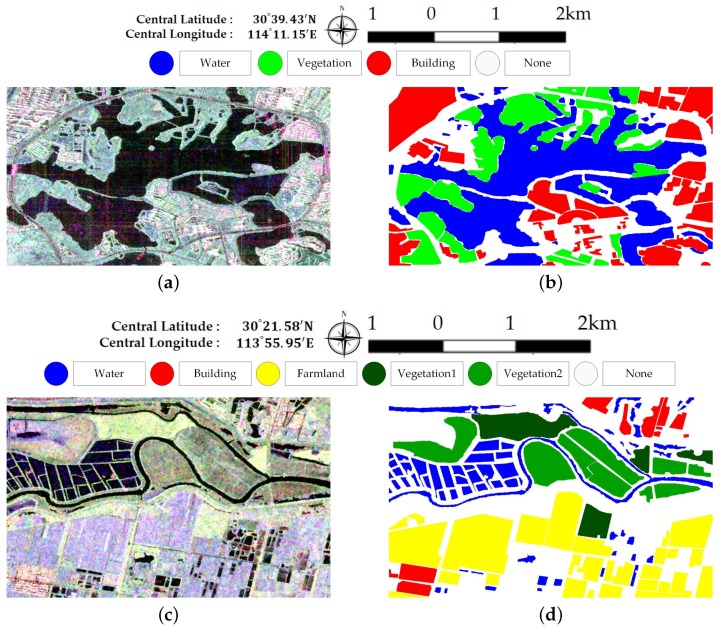
Sub-images of the Gaofen-3 PolSAR Data. (**a**) Pauli RGB image of dataset A. (**b**) The ground-truth map of (**a**). (**c**) Pauli RGB image of dataset B. (**d**) The ground-truth map of (**c**).

Figure 5 illustrates Pauli RGB images of the two data sets. Data set A has a size of 656× 1192 pixels and covers the whole region of Jinyin Lake, Wuhan. Three classes consisting of water, buildings, and vegetation, are identified, as shown in Figure 5b. Data set B has a size of 603 × 976 pixels. The study site is located at a rural region of Hannan District, Wuhan. A total of five classes are considered, which include water, building, farmland, vegetation1 and vegetation2. Vegetation areas are divided into two classes according to the backscattering responses and vegetation types, which is shown in Figure 5d. Since only Sentinel-2 optical images are available and no field survey is conducted, the exact land cover types are not identified. It is speculated that vegetation1 are shrubberies or trees with strong branches and vegetation2 involve grass or reeds that grow in wet places. Vegetation1 show stronger scattered power than vegetation2. Both ground-truth maps shown in Figure 5 are manually created based on the Sentinel-2 optical images in Figure 3. The white areas labeled “None” in both data sets are not included in the final accuracy assessment step.

### 3.2. Classification Results

In this section, initial classification results and related analysis achieved by the proposed framework from real GF-3 PolSAR data are reported. The compared classifiers include DT, RF, and SVM with radial basis function kernel. The parameters of three tree-based classifiers are set default for fair comparison. Parameter tuning is recommended for tree-based classifiers to produce better classification results. A total of *n* = 2000 labeled samples are randomly selected to train the classifiers. The number of train samples for each class is equal. Remaining samples are used for evaluation purpose. The extended Lee sigma filter with $\sigma_{v}=0.9$ and a 9 × 9 window is adopted for speckle reduction of GF-3 PolSAR data [36]. The empirical parameters of modified majority voting are set $\sigma_{1}=0.8$ and $\sigma_{2}=0.1$. *m* = 16 polarimetric features are employed as described in Section 2.1. Overall accuracy (OA) and the confusion matrix are adopted to evaluate the performance.

**Figure 6:**
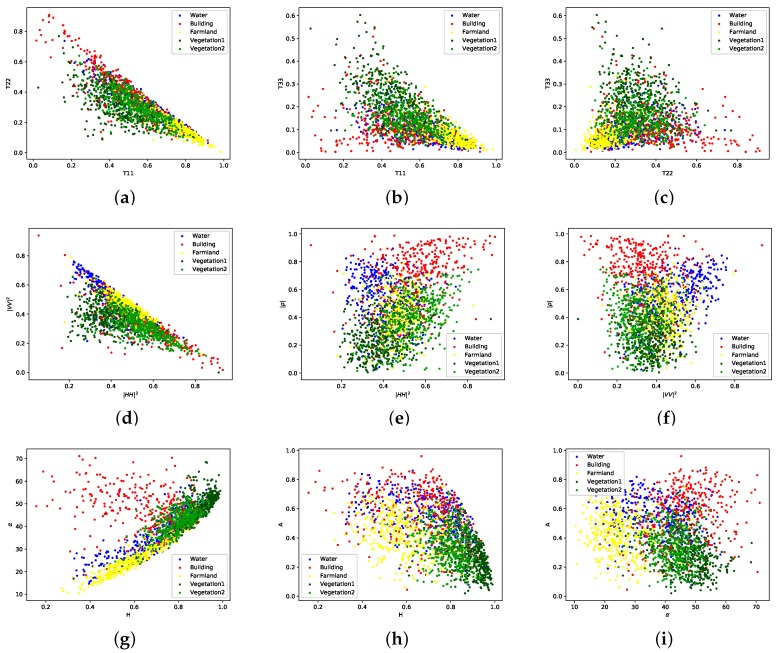

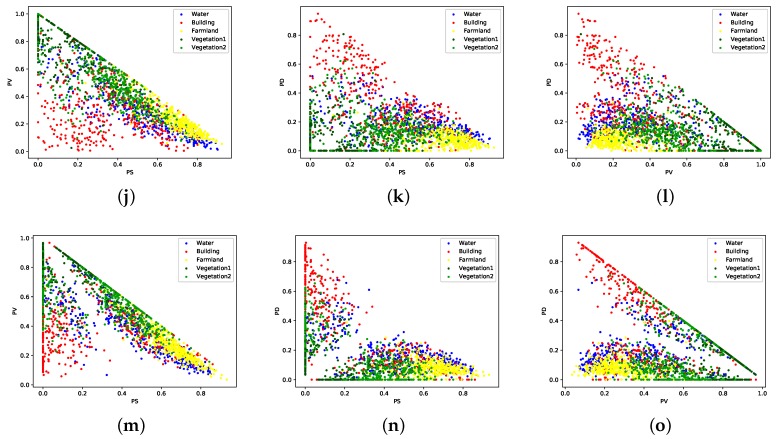
2D scatter plots of samples corresponding to different polarimetric features. Each color represents one land cover type. (**a**) $T_{11}$–$T_{22}$, (**b**) $T_{11}$–$T_{33}$, (**c**) $T_{22}$–$T_{33}$, (**d**) ${\left|HH\right|}^{2}$–${\left|VV\right|}^{2}$, (**e**) ${\left|HH\right|}^{2}$–|ρ|, (**f**) ${\left|VV\right|}^{2}$–|ρ|, (**g**) *H*–α, (**h**) *H*–*A*, (**i**) α–*A*, (**j**) $YP_{S}$–$YP_{V}$, (**k**) $YP_{S}$–$YP_{D}$, (**l**) $YP_{V}$–$YP_{D}$, (**m**) $VP_{S}$–$VP_{V}$, (**n**) $VP_{S}$–$VP_{D}$, (**o**) $VP_{V}$–$VP_{D}$. The power-related features are divided by span for visualization.

Samples of five classes consisting of water, building, farmland, vegetation1 and vegetation2, are randomly selected from data set B, image #2. Figure 6 illustrates the 2D scatter plots of different features in pairs. All the features are extracted by PolSARPro 5.1. Water and farmland show higher surface components while the volume components of both vegetation types are obvious. Figure 6g illustrates the scatter plots of five classes basically fit into the zones defined in the H/α plane [5]. However, it also shows that the used GF-3 PolSAR image shows higher values of the entropy *H*. The |ρ| value, the anisotropy *A* and the double-bounce component of buildings are higher compared to other land cover classes. The components of Yamaguchi and Van Zyl NNED decompositions reflect similar scattering mechanisms according to corresponding scatter plots. The characteristics of adopted features from GF-3 PolSAR data are basically in accordance with the theorems.

**Figure 7:**
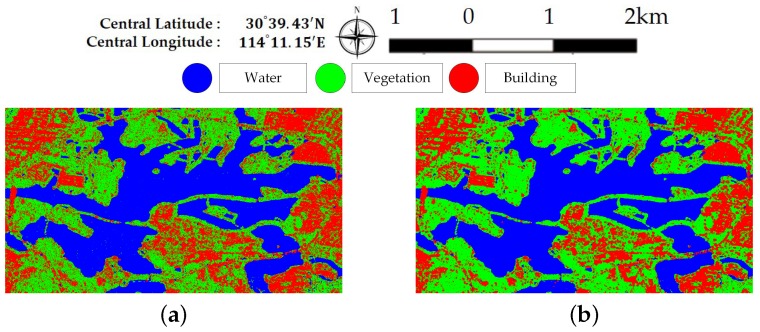

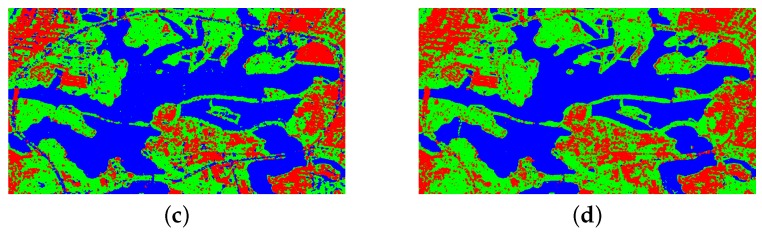
Classification maps of different classifiers on data set A. (**a**) Decision Tree. (**b**) Random Forest. (**c**) support vector machine (SVM). (**d**) XGBoost.

**Figure 8:**
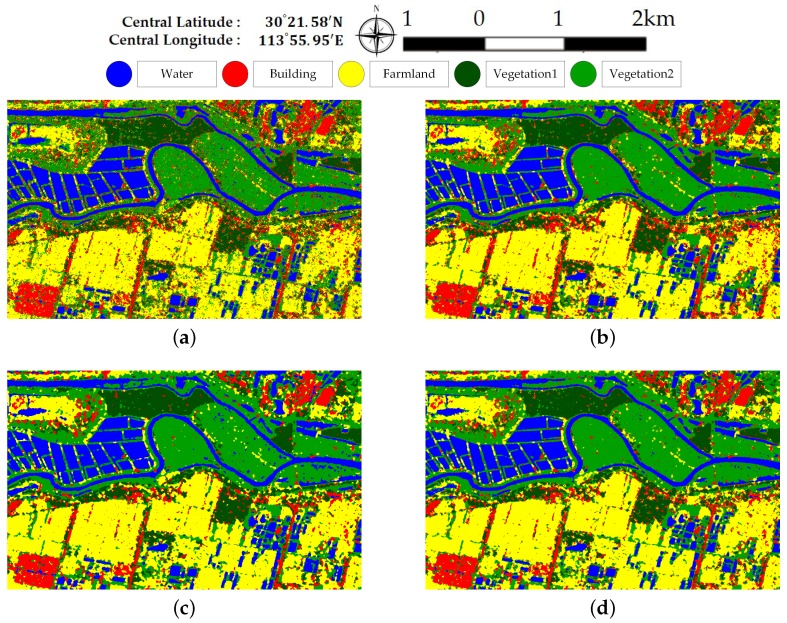
Classification maps of different classifiers on data set B. (**a**) Decision Tree. (**b**) Random Forest. (**c**) SVM. (**d**) XGBoost.

**Figure 9:**
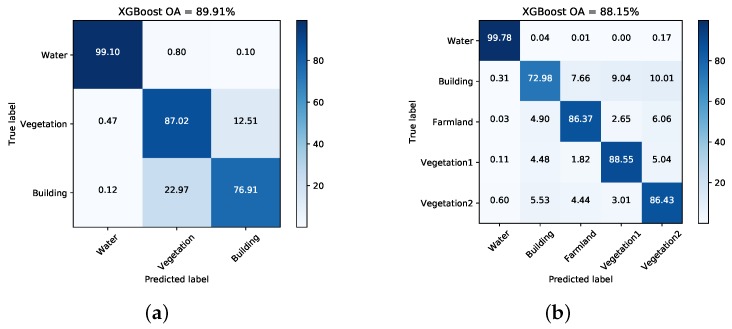
Confusion matrices acquired via XGBoost on both data sets. (**a**) Data set A. (**b**) Data set B.

Figure 7 and Figure 8 illustrate the pixel-wise classification maps on the two data sets, respectively. The OAs obtained from two data sets are shown in Table 2 and Table 3. According to the results, The OAs of DT on both data sets are worse. The other three classifiers preform well and report comparable results on both data sets. For both data sets, XGBoost performs best. From Table 3, it can be seen that the improperly classified regions mainly occur in the building area. The corresponding accuracies obtained by the four classifiers are all below 77%. Figure 9 shows the confusion matrices of both data sets, respectively. For data set A, the misclassification mainly occurs between building and vegetation types. For data set B, about 20% of the building area are misclassified as vegetation areas. The reason is that data set B covers rural region, where trees are planted around buildings. Misclassification between farmland, vegetation1 and vegetation2 results from their similarity. In all, XGBoost reports similar classification results to SVM and RF, but performs much better than DT. However, many misclassified pixels emerge in homogeneous areas for all classification maps, which badly affect the performance. Moreover, the classification results in building areas are not adequate. Some necessary steps should be taken to remove the discrete misclassified pixels and to improve the the overall performance.

### 3.3. Performance Analysis

**Figure 10:**
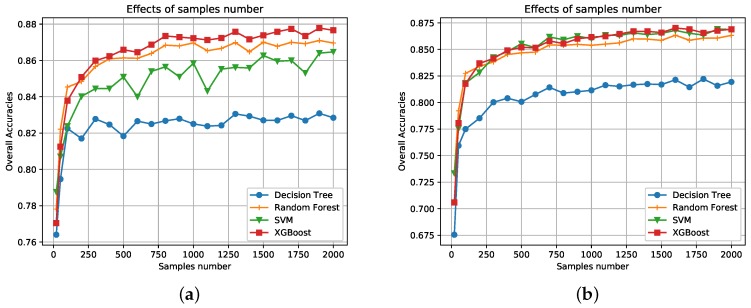
Effects of the training sample size. (**a**) Data set A. (**b**) Data set B.

To further compare performances of different classifiers, more experiments are conducted here. First, the effects of the number of training samples are examined. Figure 10 illustrates the overall classification accuracies for each classifier under different numbers of training samples. 10 to 2000 pixels of the labeled data are randomly selected as training samples. Additionally, 5000 randomly selected pixels are used for testing. Ten repeated experiments are conducted and we take the average as the final OA. Classification accuracies of both data sets generally increase as more training samples are selected. Other conclusions are same to Section 3.2. XGBoost, SVM, and RF report similar results and perform much better than DT.

**Figure 11:**
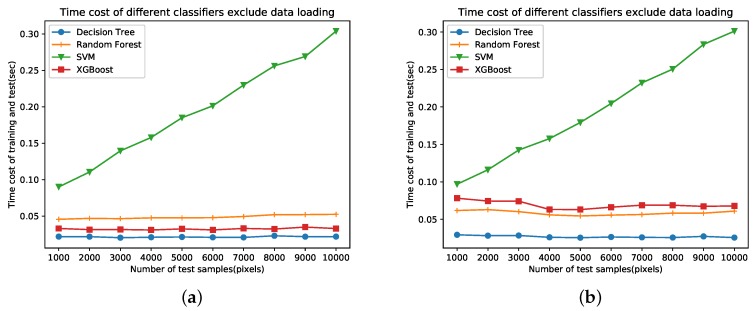
Comparison of time costs on data sets A and B. (**a**) Data set A. (**b**) Data set B.

Figure 11 shows the time costs of different classifiers. Both training samples and test samples are randomly selected. The training sample numbers is set to 2000. The number of test samples ranges from 1000 to 10,000. Accordingly, The curves of time costs on two data sets are very similar with each other. The time cost of SVM linearly increases as more samples are used. The time cost of DT is lowest but the classification result is not satisfying. SVM costs more time than XGBoost but report similar classification results. RF costs more time than XGBoost on data set A while the contrary is the case on data set B. XGBoost performs better than RF on both data sets according to Figure 10. In sum, XGBoost is most efficient in case of classification result and time cost.

### 3.4. Final Classification

**Figure 12:**
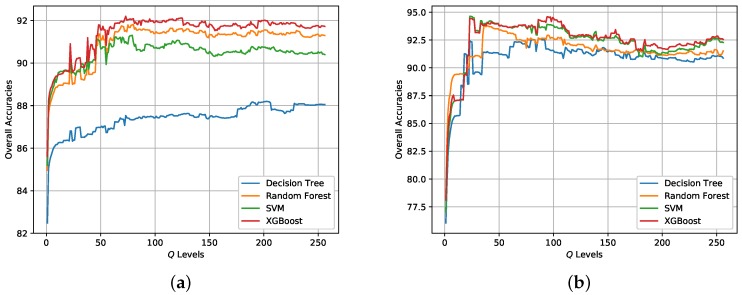
Classification results of different *Q* levels in SRM for the two data sets. (**a**) Data set A. (**b**) Data set B.

Based on the initial classification results obtained by XGBoost, the post-processing step is conducted to incorporate spatial information into the final classification map via superpixels and the modified majority voting. Superpixels are generated by SRM based on the Pauli RGB image. The parameter *Q* in SRM controls the coarseness of superpixels. The post-processing step can also be combined with other classifiers. Figure 12 shows the change of OA for different classifiers when the *Q* level increases from 1 to 256. Table 4 and Table 5 report the *Q* levels with best OAs on the two data sets, respectively. It is visible that the OAs for both data sets have improved, which demonstrates the effectiveness of incorporating spatial information through superpixels for pixel-wise classification. It can also concluded that the *Q* level has an impact on the classification result. One point is the *Q* level should not be set too small.The *Q* levels with highest OAs for the two data sets are different and should be set accordingly.

**Figure 13:**
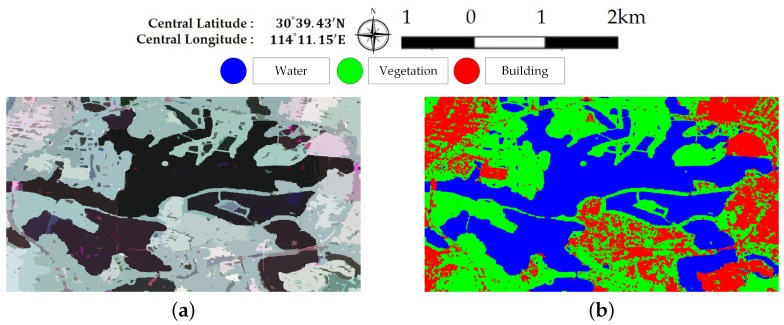
Final classification map of data set A. (**a**) Superpixels generated by SRM. (**b**) Final classification map.

**Figure 14:**
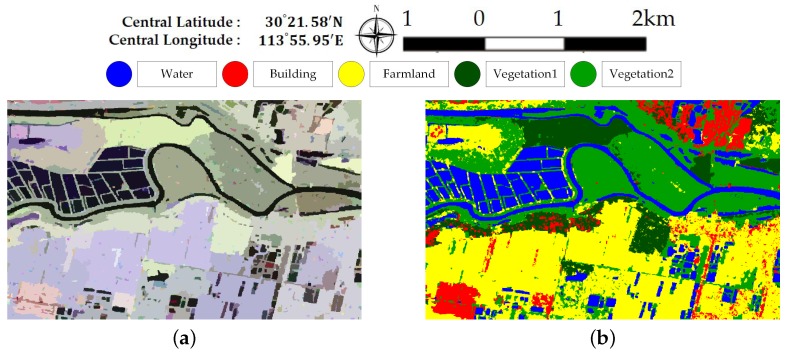
Final classification map of data set B. (**a**) Superpixels generated by SRM. (**b**) Final classification map.

**Figure 15:**
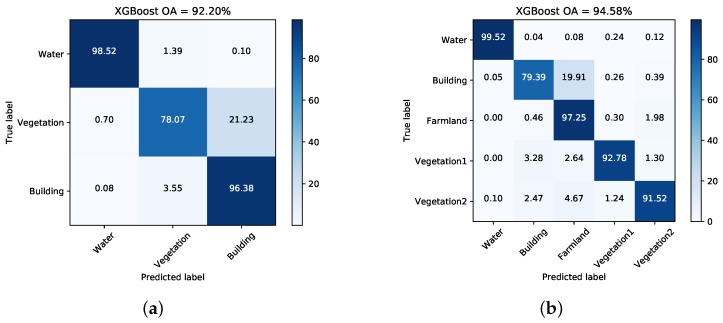
Confusion matrices with selected *Q* levels for both data sets. (**a**) Dataset A. (**b**)Dataset B.

Figure 13 and Figure 14 show the generated superpixels and the final classification maps of both data sets with selected *Q* levels, according to the OA curves in Figure 12. The specific *Q* levels can be found at Table 4 and Table 5. Compared to Figure 7 and Figure 8, the misclassified pixels in a superpixel have been removed, which helps to generate smoothed classification maps. The confusion matrices shown in Figure 15 illustrate detailed changes after the incorporation of spatial information. For data set A, the percentage at which buildings are wrongly assigned as vegetations decreases by 19.42%. The accuracy of building reaches 96.38%. The OA of data set A acquired by XGBoost is 92.20%, which is superior to other classifiers. As for data set B, the accuracies of building, farmland, and vegetations increased. Specifically, the accuracy of farmland areas in data set B is higher by 11% than that of the pixel-wise results. Overall performance and accuracies of most classes improve at the price of the misclassification between building and farmland areas. The incorporation of spatial information significantly contributes to the performance improvement. Even though SVM and XGBoost report comparable OAs according to Table 5, tree-based classifiers, especially XGBoost, are preferable in practical classification application in consideration of accuracy and time costs.

## 4. Discussions

### 4.1. Discussion on Polarimetric Features

In this section, the two GF-3 PolSAR data sets are further processed with six feature sets (FS) to find out how these features contribute to classification results. The detailed content of feature sets can be seen in Table 6. In order not to be affected by spatial information, initial classification results without the post-processing step are analyzed. Table 7 and Table 8 report the accuracies by classes and OAs with the six feature sets on both data sets. FS1 produces the lowest OAs and the lowest accuracies of building on both data sets. The H/α/A model improves the results obviously by increasing OAs by 3.96% and 5.4%, respectively. |ρ| contributes to building areas but has not benefited much to overall classification results. Both Yamaguchi and Van Zyl NNED decompositions contribute to building areas and further promote the overall performance. However, the simultaneous use of Yamaguchi and Van Zyl NNED decompositions decreases the accuracy of buildings. In all, *H*, α, and *A* are three important parameters for land cover classification. One of the Yamaguchi, Van Zyl NNED or other model-based decompositions reflecting similar scattering mechanisms is recommended.

### 4.2. Discussion on Road Type

**Figure 16:**
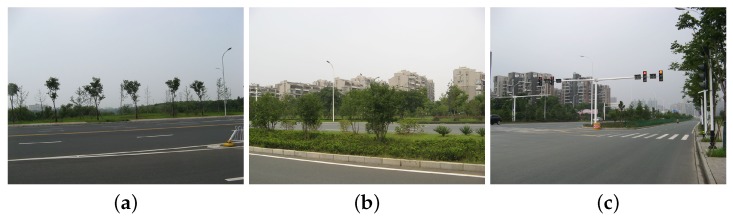
Images of roads surrounding the Jinyin Lake in data set A. **a**, **b**, and **c** are different images of roads. The images were taken on 16 August 2017.

**Figure 17:**
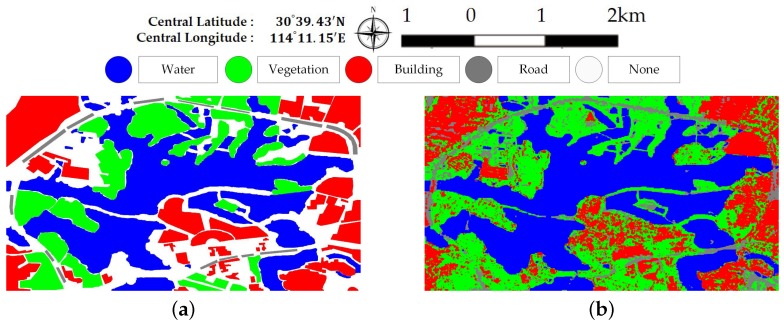
Ground truth map and final classification map of data set A with four classes. (**a**) Ground trup map. (**b**) Final classification map.

**Figure 18:**
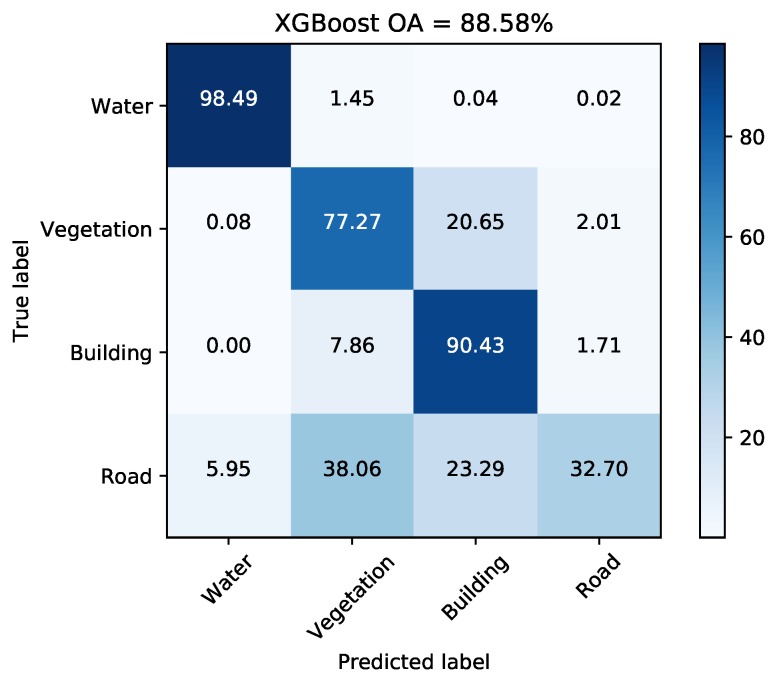
Confusion matrix for data set A with four classes.

In data set A, the roads surrounding Jinyin Lake often appear as vegetation types. This is probably due to the lack of training samples since current land cover classes do not contain roads. Another possible reason is that trees are planted along the roads as shown in Figure 16. Here, some training sites on the road around Jinyin Lake are added into the original ground truth map. Figure 17 shows the updated ground truth map and final classification map with the road type. Some parts of roads are extracted. Some none-road areas are unexpectedly misclassified as roads. Based on the confusion matrix shown in Figure 18, the accuracy of road is only 32.70% and the accuracies acquired by the other three compared classifiers are 29.20% (DT), 34.56% (RF), and 21.21% (SVM). The majority of misclassification can be found in vegetation and building areas for trees and buildings alongside roads. The classification methods are unsuitable for the extraction of roads or other line targets.

### 4.3. Discussion on More Complex Land Cover Classification System

**Figure 19:**
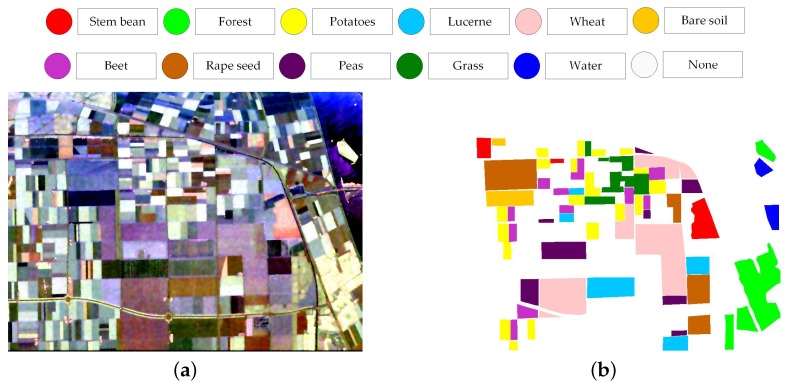
Pauli RGB image (**a**) and ground truth map (**b**) of AIRSAR Flevoland data set.

To further evaluate the performance on the data set with a more complex land cover classification system, the AIRSAR Flovland data set is tested by the proposed classification framework. The scene covers over Flevoland, the Netherland with an image size of 750 × 1024 pixels. With well-established ground truth map, the AIRSAR Flevoland data set has been widely used in land use classification since [38]. The land cover classification system consists of eight crop classes and three other classes of bare soil, water, and forest, which is shown in Figure 19. In consideration of the homogeneity of this data set, the empirical parameters of the modified majority voting are set $\sigma_{1}=0.5$ and $\sigma_{2}=0.4$. The extended Lee sigma filter is adopted and other parameter settings are same to Section 3.

**Figure 20:**
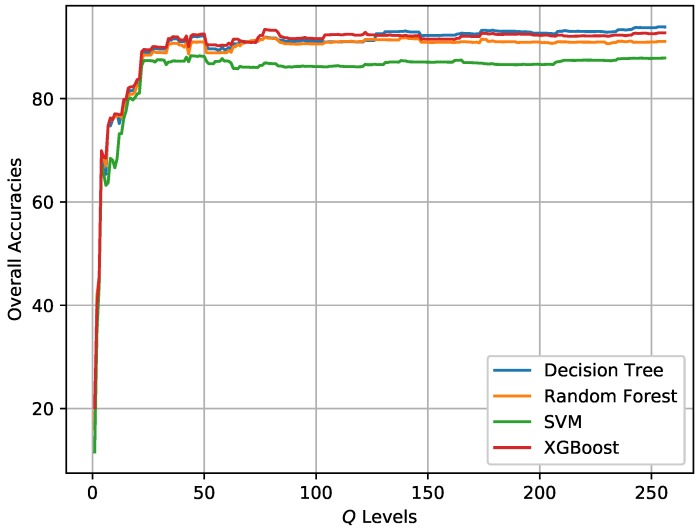
Classification results of different *Q* levels in SRM for AIRSAR Flevoland data set.

**Figure 21:**
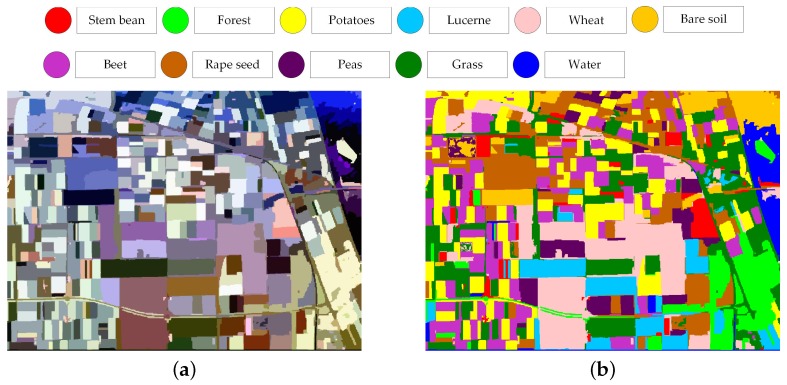
Final classification map of AIRSAR Flevoland data set. (**a**) Superpixels generated by SRM. (**b**) Final classification map.

**Figure 22:**
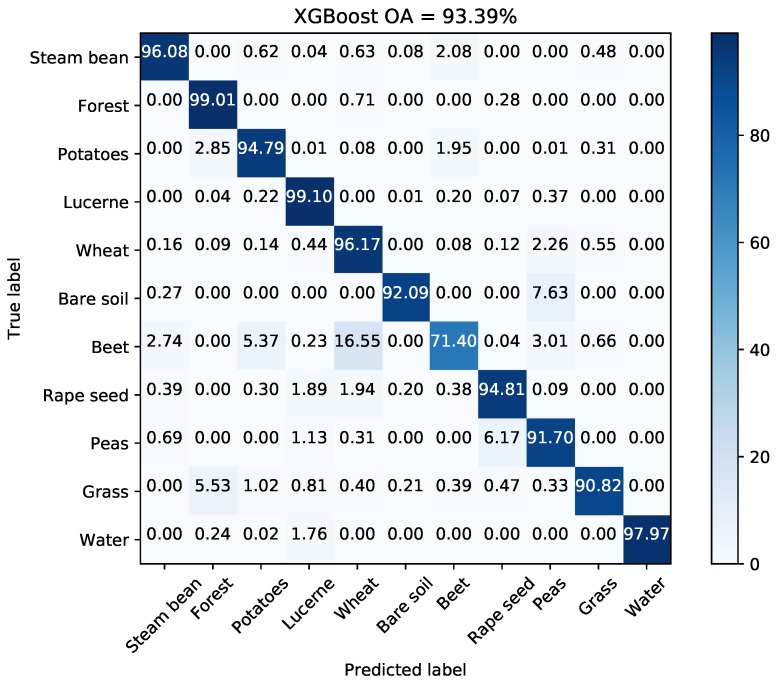
Confusion matrix with the selected *Q* level for AIRSAR Flevoland data set.

Figure 20 illustrates the change of OA with different *Q* levels. Table 9 reports the *Q* level with best OA for different classifiers. The OAs acquired by methods proposed by Lee et al. [38], Tao et al. [10], and Qin et al. [11] are also reported in Table 9 since the same data set has been used and land cover classes are same. Lee et al. used an maximum likelihood classifier based on the Wishart distribution for all polarization combinations to quantitatively assess classification results [38]. Tao et al. developed a classification method with selected hidden polarimetric features in the rotation domain and a SVM/DT classifier. The overall classification accuracy of DT with a window size of 9×9 is shown in Table 9 in view of better performance and fair comparison [10]. Qin et al. developed an object-oriented classification method using restricted Boltzmann machines. The best OA of the proposed RBM-Adaboost with 29 units and 88 learners is 96.15% [11]. According to the results, tree-based classifiers surpass SVM by 3%. The OA of XGBoost is higher by 10% than that of Lee’s method. Figure 21 shows the generated superpixels and the final classification map acquired by XGBoost. The corresponding confusion matrix is shown in Figure 22. The accuracies of most classes except for beet, are above 90%. Therefore, the proposed method is competitive with other benchmark methods.

## 5. Conclusions

In this paper, we proposed an efficient classification framework for GF-3 PolSAR images. With quick initial classification and incorporation of spatial information, the proposed method is hopefully able to classify an entire GF-3 PolSAR image quickly and accurately. We also analyzed the characteristics of features from GF-3 PolSAR data and how the adopted features contribute to classification results. Preliminary experimental results on real GF-3 PolSAR images and the AIRSAR Flevoland PolSAR data have demonstrated their own advantages. XGBoost showed comparable classification results with less time consumed. Spatial information turned out to be an important factor for PolSAR image classification and accounted for the accuracy improvements achieved in the post-processing step via superpixel-based modified majority voting. The results also showed that the quality of GF-3 PolSAR data was adequate enough for land cover classification and other potential usages.

Further improvement is still required. Analysis about more palarimetric parameters extracted from Gaofen-3 data [39] and how to choose essential polarimetric features need to be investigated. The generation of adequate superpixels is worth further exploration. Future efforts should also be devoted to implementing the classification framework in C++, which will further improve efficiency.

## Figures and Tables

**Table 1:** Data description of the used GF-3 PolSAR images.

Parameter	Image #1	Image #2
Imaging time	29 May 2017	1 April 2017
Polarization	AHV	AHV
Product type	SLC	SLC
Imaging mode	QPSI	QPSI
Satellite direction	Ascending	Ascending
Nominal resolution (m)	8	8
Spatial resolution [Range × Azimuth] (m)	5.53 × 2.25	5.20 × 2.25
Image size [Range × Azimuth] (pixel)	7469 × 6210	7856 × 6805
Incidence angles (${}^{{}^{\circ}}$)	35.29–36.99	33.69–35.61

**Table 2:** Classification accuracies on data set A: by classes and overall accuracy (%).

Classifiers	Water	Vegetation	Building	OA
Decision Tree	98.90	72.76	76.66	86.36
Random Forest	99.23	85.84	75.36	89.26
SVM	99.66	87.69	72.39	89.06
XGBoost	99.10	87.02	76.91	89.91

**Table 3:** Classification accuracies on data set B: by classes and overall accuracy (%).

Classifiers	Water	Building	Farmland	Vegetation1	Vegetation2	OA
Decision Tree	99.42	70.34	79.27	81.65	78.70	82.49
Random Forest	99.76	76.58	83.75	87.38	84.03	86.64
SVM	99.83	72.25	86.12	89.92	85.54	87.96
XGBoost	99.78	72.98	86.37	88.55	86.43	88.15

**Table 4:** *Q* levels with the best overall accuracies (OAs) on data set A.

	Decision Tree	Random Forest	SVM	XGBoost
Best OA (%)	88.19	91.81	91.30	92.20
*Q* level	203	80	79	73

**Table 5:** *Q* levels with best OAs on data set B.

	Decision Tree	Random Forest	SVM	XGBoost
Best OA (%)	92.68	93.77	94.63	94.58
*Q* level	86	39	23	95

**Table 6:** Detailed content of feature sets.

Feature Set	Polarimetric Features
FS1	${\left|HH\right|}^{2}$, ${\left|VV\right|}^{2}$, $T_{11}$, $T_{22}$, $T_{33}$
FS2	FS1, *H*, α, *A*
FS3	FS2, |ρ|
FS4	FS3, $VP_{S}$, $VP_{V}$, $VP_{D}$
FS5	FS3, $YP_{S}$, $YP_{V}$, $YP_{D}$, $YP_{C}$
FS6	All the *m* = 16 features

**Table 7:** Accuracies by classes and OAs with different feature sets on data set A (%).

Feature Set	Water	Vegetation	Building	OA
FS1	98.54	76.94	63.44	83.42
FS2	98.86	84.15	70.80	87.38
FS3	98.31	81.43	73.78	87.32
FS4	99.30	85.58	78.46	90.11
FS5	98.56	83.56	80.43	89.84
FS6	99.10	87.02	76.91	89.91

**Table 8:** Accuracies by classes and OAs with different feature sets on data set B (%).

Feature Set	Water	Building	Farmland	Vegetation1	Vegetation2	OA
FS1	99.39	50.85	79.12	78.43	80.96	81.20
FS2	99.43	69.73	84.80	86.81	84.55	86.60
FS3	99.30	69.73	85.71	88.40	82.10	86.62
FS4	99.77	74.59	85.95	87.67	87.40	88.19
FS5	99.48	72.41	86.76	89.29	85.29	88.06
FS6	99.78	72.98	86.37	88.55	86.42	88.15

**Table 9:** OAs of different classifiers on AIRSAR Flevoland data set.

	Decision Tree	Random Forest	SVM	XGBoost	Lee [38]	Tao [10]	Qin [11]
Best OA (%)	93.90	91.87	88.31	93.39	81.63	94.58	96.15
*Q* level	253	77	44	77	–	–	–
